# Prognostic factors of perioperative FLOT regimen in operable gastric and gastroesophageal junction tumors: real-life data (Turkish Oncology Group)

**DOI:** 10.55730/1300-0144.5404

**Published:** 2022-05-22

**Authors:** Cihan EROL, Abdullah SAKİN, Tuğba BAŞOGLU, Ercan ÖZDEN, Devrim ÇABUK, Mutlu DOĞAN, Berna ÖKSÜZOĞLU, Hasan Çağrı YILDIRIM, İrem ÖNER, Melek KARAKURT ERYILMAZ, Özgecan DÜLGAR, Dinçer AYDIN, Neslihan DOĞAN, Miraç ÖZEN, İlhan HACIBEKİROĞLU, Nuriye ÖZDEMİR, Fatih GÜRLER, Nail PAKSOY, Senem KARABULUT, Asude AKSOY, Mutlu HIZAL, Seda KAHRAMAN, Erdem ŞEN, Semra PAYDAŞ, Ebru ÇILBIR, Feyza FIRAT, Nadiye AKDENİZ, Melike ÖZÇELİK, Abdilkerim OYMAN, Naziyet KÖSE BAYTEMÜR, Ramazan ACAR, Elvina ALMURADOVA, Bülent KARABULUT, Teoman ŞAKALAR, Hacı ARAK, Ezgi DEĞERLİ, Sema TÜRKER, Özkan ALAN, Özlem ER, Elif ŞENOCAK TAŞÇI, Nazan DEMİR, Eyyüp ÇAVDAR, Serdar TURHAL, Didem ŞENER DEDE, Muhammed Bülent AKINCI, Bülent YALÇIN, Fulden YUMUK, Şuayib YALÇIN, Mehmet Ali Nahit ŞENDUR

**Affiliations:** 1 Department of Medical Oncology, Faculty of Medicine, Ankara Yıldırım Beyazıt University, Ankara, Turkey; 2 Department of Medical Oncology, Faculty of Medicine, Van Yüzüncü Yıl University, Van, Turkey; 3 Department of Medical Oncology, School of Medicine, Marmara University, İstanbul, Turkey; 4 Department of Medical Oncology, Faculty of Medicine, Kocaeli University, Kocaeli, Turkey; 5 Department of Medical Oncology, Ankara Dr. Abdurrahman Yurtaslan Oncology Training and Research Hospital, Ankara, Turkey; 6 Department of Medical Oncology, Faculty of Medicine, Hacettepe University, Ankara, Turkey; 7 Department of Medical Oncology, Konya Meram State Hospital, Konya, Turkey; 8 Department of Medical Oncology, Meram Faculty of Medicine, Necmettin Erbakan University, Konya, Turkey; 9 Department of Medical Oncology, Göztepe Training and Research Hospital, İstanbul Medeniyet University, İstanbul, Turkey; 10 Department of Medical Oncology, Derince Training and Research Hospital, Kocaeli, Turkey; 11 Department of Medical Oncology, Prof. Dr. A. İlhan Özdemir Education and Research Hospital, Giresun University, Giresun, Turkey; 12 Department of Medical Oncology, Faculty of Medicine, Sakarya University, Sakarya, Turkey; 13 Department of Medical Oncology, Faculty of Medicine, Gazi University, Ankara, Turkey; 14 Department of Medical Oncology, Institute of Oncology, İstanbul University, İstanbul, Turkey; 15 Department of Medical Oncology, Faculty of Medicine, Fırat University, Elazığ, Turkey; 16 Department of Medical Oncology, Ankara City Hospital, Ankara, Turkey; 17 Department of Medical Oncology, Çanakkale Mehmet Akif Ersoy State Hospital, Çanakkale, Turkey; 18 Department of Medical Oncology, Faculty of Medicine, Çukurova University, Adana, Turkey; 19 Department of Medical Oncology, Dışkapı Training and Research Hospital, Ankara, Turkey; 20 Department of Medical Oncology, Faculty of Medicine, İnönü University, Malatya, Turkey; 21 Department of Medical Oncology, Adıyaman Training and Research Hospital, Adıyaman, Turkey; 22 Department of Medical Oncology, Ümraniye Education and Research Hospital, İstanbul, Turkey; 23 Department of Medical Oncology, Memorial Ankara Hospital, Ankara, Turkey; 24 Department of Medical Oncology, Gülhane Education and Research Hospital, Ankara, Turkey; 25 Department of Medical Oncology, Faculty of Medicine, Ege University, İzmir, Turkey; 26 Department of Medical Oncology, Kahramanmaraş Necip Fazıl City Hospital, Kahramanmaraş, Turkey; 27 Department of Medical Oncology, Faculty of Medicine, Gaziantep University, Gaziantep, Turkey; 28 Department of Medical Oncology, Cerrahpaşa School of Medicine, İstanbul University, İstanbul, Turkey; 29 Department of Medical Oncology, Zonguldak Atatürk State Hospital, Zonguldak, Turkey; 30 Department of Medical Oncology, Tekirdağ State Hospital, Tekirdağ, Turkey; 31 Department of Medical Oncology, Acıbadem Maslak Hospital, İstanbul, Turkey; 32 Department of Medical Oncology, Faculty of Medicine, Osmangazi University, Eskişehir, Turkey; 33 Department of Medical Oncology, Faculty of Medicine, Tekirdağ Namık Kemal University, Tekirdağ, Turkey; 34 Department of Medical Oncology, Anadolu Medical Center, Kocaeli, Turkey

**Keywords:** FLOT chemotherapy, perioperative treatment, gastric cancer, GEJ tumor, prognostic factor

## Abstract

**Background/aim:**

Perioperative FLOT regimen is a standard of care in locally advanced operable gastric and GEJ adenocarcinoma. We aimed to determine the efficacy, prognostic factors of perioperative FLOT chemotherapy in real-life gastric and GEJ tumors.

**Materials and methods:**

The data of patients who were treated with perioperative FLOT chemotherapy were retrospectively analyzed from 34 different oncology centers in Turkey. Baseline clinical and demographic characteristics, pretreatment laboratory values, histological and molecular characteristics were recorded.

**Results:**

A total of 441 patients were included in the study. The median of age our study population was 60 years. The majority of patients with radiological staging were cT3-4N(+) (89.9%, n = 338). After median 13.5 months (IQR: 8.5–20.5) follow-up, the median overall survival was NR (95% CI, NR to NR), and median disease free survival was 22.9 (95% CI, 18.6 to 27.3) months. The estimated overall survival at 24 months was 62%. Complete pathological response (pCR) and near pCR was achieved in 23.8% of all patients. Patients with lower NLR or PLR have significantly longer median OS (p = 0.007 and p = 0.033, respectively), and patients with lower NLR have significantly longer median DFS (p = 0.039), but PLR level did not affect DFS (p = 0.062). The OS and DFS of patients with better ECOG performance scores and those who could receive FLOT as adjuvant chemotherapy instead of other regimens were found to be better. NLR was found to be independent prognostic factor for OS in the multivariant analysis. At least one adverse event reported in 57.6% of the patients and grade 3–4 toxicity was seen in 23.6% patients.

**Conclusion:**

Real-life perioperative FLOT regimen in operable gastric and GEJ tumors showed similar oncologic outcomes compared to clinical trials. Better performance status, receiving adjuvant chemotherapy as same regimen, low grade and low NLR and PLR improved outcomes in real-life. However, in multivariate analysis, only NLR affected OS.

## 1. Introduction

Gastric and gastroesophageal junction (GEJ) adenocarcinoma is among one of the cancers with poor prognosis. Application of multimodal treatment protocols contributes to prognosis by providing local and systemic tumor control as well as increasing surgical resectability in patients with locally advanced gastric and GEJ adenocarcinoma. Survival benefit is tried to be achieved with adjuvant and neoadjuvant chemotherapy and radiotherapy. However, the results are still not satisfactory. The 5-year overall survival (OS) rate with perioperative chemotherapy is between 36% and 38% in operable gastric cancers [1,2]. The MAGIC study is the cornerstone for perioperative chemotherapy [2]. In this study, perioperative ECF (Epirubicin, cisplatin, 5-FU) chemotherapy was compared with surgery alone. Compared to the surgical group alone, the perioperative chemotherapy group had significantly higher median OS and progression-free survival (PFS) (hazard ratio for death, 0.75; 95% CI; p = 0.009, and hazard ratio for progression, 0.66; 95% CI; p < 0.001). FLOT chemotherapy regimen (5-FU, leucovorin, oxaliplatin, docetaxel) has been considered in perioperative treatment because of its better tolerability and response rates in metastatic disease. It was the choice of perioperative chemotherapy protocol since 2009. The FLOT4-AIO Phase 2/3 study is a randomized controlled study evaluating the efficacy and safety of perioperative FLOT therapy in locally advanced and operable gastric and gastroesophageal junction tumors [3]. In this study, in which approximately 700 patients were randomized 1:1, the median OS compared to the ECF regimen was 50 months and 35 months in favor of the FLOT regimen with HR:0.77 (95% CI: 0.63 to 0.94). After this dramatic benefit, perioperative FLOT chemotherapy has become a standard of care in gastric and GEJ adenocarcinoma at cT2 and higher stages.

Clinical trial results and real-life outcomes may differ from each other. Because the patients included in the clinical trial are highly selective patients and results can be found better than in real-life. Therefore, real-life data have an important place in confirming clinical trials. The next step after determining the standard treatment for a disease is to determine which patients will benefit more from this treatment. By determining predictive and prognostic factors, patient selection can be made more accurately, and which patients’ group will benefit from the treatment can be predicted and the best treatment option can be offered.

There are several prognostic factors for gastric cancer [4, 5]. The Memorial Sloan Kettering Cancer Center nomogram is a model predicting survival for gastric cancer included age, sex, primary tumor site, tumor size, histology, number of lymph nodes resected (positive and negative), and depth of invasion [4]. It was identified 23 potentially relevant prognostic factors and 15 predictive factors for gastric cancer in a systematic review and metaanalysis. These included prognostic factors such as T stage, N stage, weight, hemoglobin value, weight loss, and predictive factors such as age, sex, T stage, N stage, HER2 overexpression, and histology [6].

There are many studies that have showed inflammation is the main cause of tumorigenesis [7]. Studies have shown that inflammation can initiate cancer [8]. Neutrophils, platelets and lymphocytes have important roles in tumor-associated inflammation. Neutrophils and platelets increase inflammation, while lymphocytes can produce inhibitory cytokines and reduce tumor cell motility. Therefore, decrease in lymphocyte count with increase in neutrophile and platelet count may lead to less immunological response against malignancies [9]. Neutrophil-lymphocyte ratio (NLR) and platelet-lymphocyte ratio (PLR) have been shown to have significant value, especially in gastrointestinal and lung cancers [10, 11]. However, the prognostic value of NLR and PLR is unclear in operable gastric and GEJ cancers receiving perioperative FLOT chemotherapy.

In this study, we aimed to determine the efficacy of perioperative FLOT chemotherapy as well as its prognostic factors, including NLR and PLR, in real-life operable gastric and gastroesophageal junction tumors in Turkey.

## 2. Materials and methods

The patients with gastric and gastroesophageal junction tumors who were treated with perioperative FLOT chemotherapy were retrospectively analyzed. The data of the patients was collected from 34 different oncology centers in Turkey. All patients who started FLOT chemotherapy as neoadjuvant therapy were included in the study. FLOT regimen includes 5-FU, leucovorine, oxaliplatin and docetaxel. They are applied intravenously; oxaliplatin 85 mg/m^2^, docetaxel 50 mg/m^2^, and leucovorin 200 mg/m^2^ on day 1 and then 5-FU 2600 mg/m^2^ 24 h infusion, every 2 weeks [3]. The standard perioperative treatment was four cycle preoperative and four cycle postoperative applications.

This study was planned as a Turkish Oncology Group (TOG) study and data were collected from medical oncology clinics across Turkey. We conducted this study according to the Declaration of Helsinki (1964) and all its subsequent amendments. Each investigator provided signed, written, informed consent before enrolment. And we started the study after it was found ethically appropriate at the Ankara City Hospital Ethics Committee meeting on 16/06/2021, with the decision number E2-21-617.

### 2.1. Data acquisition

The patients baseline clinical and demographic characteristics, pretreatment laboratory values (complete blood count, albumin value, tumor marker levels), clinical and pathological stage, and histological and molecular characteristics were recorded in the database. Treatment characteristics (response and toxicity) were noted. NLR was calculated by dividing the neutrophil count by the lymphocyte count, and PLR was calculated by dividing the platelet count by the lymphocyte count. Based on the median value of NLR and PLR (2.8 for NLR and 167.7 for PLR), it was divided into high and low. Values below the median value were grouped as low, and others were grouped as high. Disease progression and survival information during or after treatment were collected and used for survival analyses.

### 2.2. Inclusion criteria and outcomes

Patients aged 18 years and older, who were diagnosed with operable gastric or GEJ tumor histopathologically, and who started perioperative FLOT chemotherapy, were included in the study regardless of their operation status. Patients diagnosed between 01 January 2017 and 31 December 2020 were screened. Patients who received at least one treatment cycle for perioperative purposes were included in the analysis.

The primary endpoints were overall survival (OS) and disease free survival (DFS). OS was defined as the time elapsed between initiation of treatment and death from any cause. DFS was defined as the time elapsed between initiation of therapy and radiological disease progression or death from any cause if there was no progression. Secondary endpoints were objective response rate (ORR), pathologic complete response (pCR) rate, and adverse events. Objective response rate was defined as patients with complete or partial response radiologically. Adverse events (AEs) were evaluated according to CTCAE v4.03.

### 2.3. Statistical analysis

The results of study were obtained through the analysis of our retrospective database. Statistical analysis was performed using IBM SPSS statistics, Version 25.0 (SPSS Inc., Chicago, IL, USA). Continuous variables were summarized with mean, median, standard deviation, and interquartile range. Categorical variables were summarized with absolute frequency and percentages. Differences between groups were evaluated with the chi-square test. Quantitative values were expressed as medians with range, and differences were measured using the Mann–Whitney U test. Survival was univariately analyzed by the Kaplan–Meier method with a log-rank test for the comparison of subgroups. Logistic regression analysis was used to analyze the effect of multiple variables on survival. p value < 0.05 was considered statistically significant.

## 3. Results

### 3.1. Patients and disease characteristics

A total of 441 patients data were analyzed in the study. The median age of our study population was 60 years (18–85). The percentages of the disease-subtype according to tumor location were 46,3% for GEJ, 26,3% corpus, 24% antrum, 3,4% fundus. Of the 338 patients with radiological staging information, 0.9% were cT1-2/N(−), 4.7% cT1-2/N(+), 4.4% cT3-4/N(−) and 89.9% cT3-4/N (+). Baseline patient, disease and treatment characteristics summarized in Table 1.

### 3.2. Treatment characteristics and survival outcomes

Median number of preoperative and adjuvant FLOT cycles are 4 (range: 1–12) and 4 (range: 0–8), respectively (Table 1). While 93.7% of the patients could undergo surgery, the R0 resection rate was 86.6% in the data available (n = 402). Of the patients, 6.3% could not undergo surgery. Fifteen percent (66) of patients received extended neoadjuvant FLOT regimen more than 4 cycles. The R0 resection rate in these patients is 69.7%, significantly lower than in other patients. Twenty-six patients received more than 4 cycles of FLOT as adjuvant therapy.

**Figure 1:**
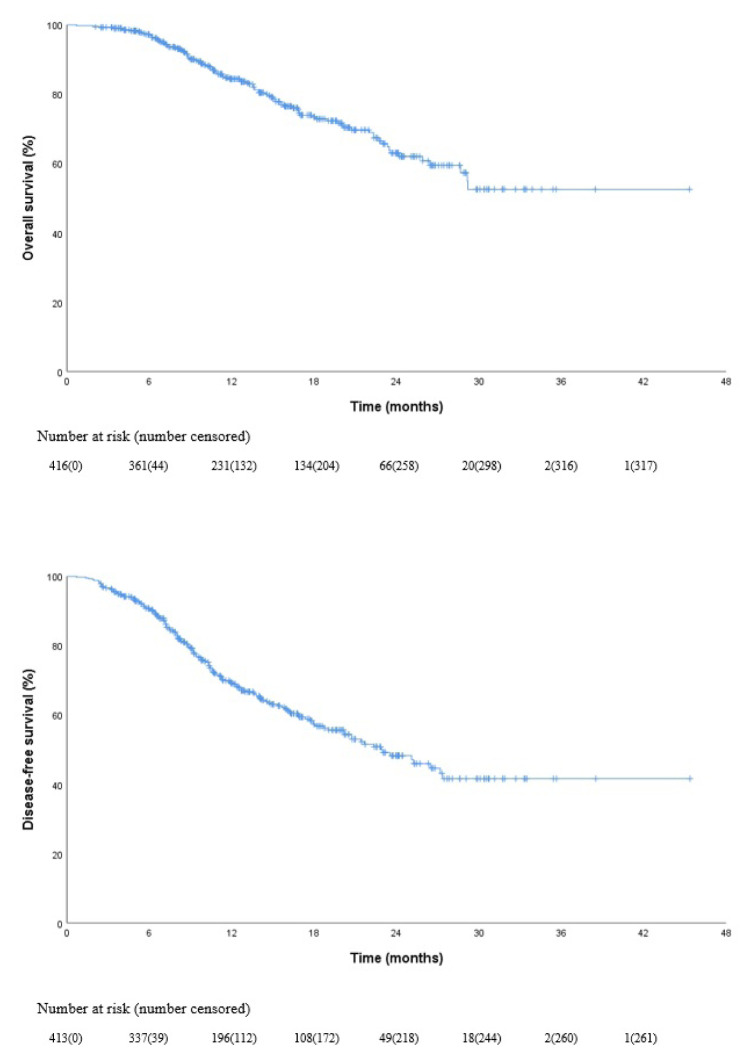
Kaplan-Meier curves for overall survival and disease-free survival in all patients.

After median 13.5 months (IQR: 8.5–20.5) follow-up the estimated median OS was not reached (NR) (95% CI, NR to NR), and median disease free survival was 22.9 (95% CI, 18.6 to 27.3) months (Figure 1). The estimated OS rate at 24 months was 62% (Figure 1).

### 3.3. Prognostic factors

Complete pathological response (pCR) and near pCR was achieved in 23.8% of all patients. We identified that pCR is a predictor of improved overall and disease free survival (p = 0.033, p = 0.030 for OS and DFS, respectively).

**Figure 2:**
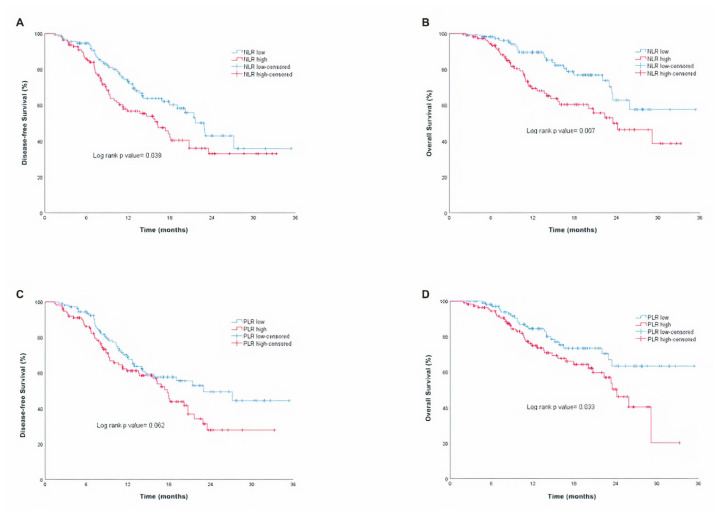
Kaplan-Meier curves for disease-free survival and overall survival according to NLR high and low (A–B), PLR high and low (C–D).

Patients with low NLR or PLR have a longer OS (p = 0.007 and p = 0.033, respectively), and patients with low NLR have a longer DFS (p = 0.039), but PLR level did not affect DFS (p = 0.062) (Figures 2A–2B and 2C–2D). The OS and DFS of patients with better ECOG performance scores and those who could receive FLOT as adjuvant chemotherapy instead of other regimens were found to be better. The effects of the variables on OS and DFS summarized with details in Table 2.

ORR was found to be 58.7% after neoadjuvant therapy in patients whose radiological evaluation could be obtained (n = 133) (Table 3). Relaps occurred after a median of 9.2 months (IQR: 6.2–12.7). The major recurrence site was peritoneal carcinomatosis (52.5%).

Multivariant logistic regression analysis was performed to identify the factors that actually effect survival within the variables. Variables with a p value of less than 0.25 on OS were included in the analysis. However, the ECOG performance score, adjuvant chemotherapy status, and clinical stage were excluded from the analysis because their distribution was not normal. NLR was found to be independent prognostic factor for OS in the multivariant analysis of NLR, PLR, grade, CEA and CA19-9 level (Table 4).

### 3.4. Safety

At least one adverse event reported in 57.6% of the patients and grade 3–4 toxicity was seen in 23.6% patients. While the most common side effect was neutropenia (26.1% any grade, 11.8% grade 3–4), fatigue was the other common side effect (9.5% any grade). The most common adverse events summarized in Table 5.

## 4. Discussion

Surgery involving D2 lymph node dissection and R0 resection is the only curative treatment option for gastric and GEJ cancers. Since there is no specific screening program in most countries, more than half of the patients are diagnosed at locally advanced stage [12]. Multimodal treatment options are used to increase the rate of curative treatment. Perioperative chemotherapy has been used for many years in locally advanced gastric and GEJ tumors because of its survey advantage by downstaging the tumor and reducing the risk of local and distant relapses by eradicating the micrometastatic disease. For this purpose, platinum and anthracycline-based chemotherapy was used most frequently [1, 2].

In the FLOT4-AIO trial, the MAGIC regimen was compared with the taxane-containing FLOT regimen. The median OS and DFS was 50 and 30 months, respectively [3]. And OS at 2 years was 68% and ≤ypT1 was 25%. With this study, 5-year survival increased from 35%–40% to 45%. In our study, median OS was not reached and median DFS was 22.9 months. Estimated OS rate at 2 years was 62% and ≤ypT1 (tumor invades the submucosa following preoperative chemotherapy) was 26.3%. One reason for the slightly lower survival compared to the FLOT4-AIO trial was the inclusion of clinically worse patients (a real-life classic) and advanced stage. While the rate of patients with an ECOG performance score of 0–1 in our study was 90%, this rate was 99% in the FLOT4-AIO study. The other reason of lower survival was the higher stage of disease. In pivotal trial of FLOT [3], the rate of patients with clinically T3-4 was 83% and node positive patients was 78%. These rates were over 94% for both in our study. On the other hand, it would be expected that more advanced and clinically worse patients may undergo less surgery; however, a similar rate of surgery was performed in our study and the FLOT4-AIO trial (94%). And patients had similar pCR and near pCR rates (25% vs. 23.8%) in all patients.

In the univariate analysis, we determined that better ECOG performance score, low grade, continuing adjuvant chemotherapy as FLOT, and low NLR improved OS and DFS. PLR did not affect DFS, but patients with low PLR had longer OS. In the multivariate analysis, we determined that NLR was independent predictive factors for OS.

NLR is a well-known prognostic factor in multiple tumors [13, 14]. Higher NLR is associated with worse survival outcomes [15, 16]. In a metaanalysis of breast cancer, it was shown that NLR was a prognostic factor for overall survival, independently of tumor stage [17]. Similarly with NLR, PLR can be used as a marker for inflammation. Prognostic features have been demonstrated in many tumors [15, 16]. In our study, lower NLR and PLR were found to be associated with better survival, similar to previous studies [15, 16, 18]. This result is important; however, hematological values of patients may change after neoadjuvant treatment. In a study published in 2020 including gastric cancer patients receiving neoadjuvant chemotherapy, higher NLR was related with worse overall survival when evaluated before neoadjuvant treatment [19]. In that trial, in the multivariate analysis of preneoadjuvant treatment values, no variable was found to be an independent prognostic factor. In addition, in the combined analysis of inflammatory markers before and after neoadjuvant treatment, NLR lost its prognostic feature.

In this analysis, receiving FLOT as adjuvant chemotherapy appeared to be more beneficial for survival than others. There are conflicting data on this subject. However, an important observational study on this subject revealed that it is important to complete perioperative chemotherapy. The 5-year survival rate in patients who received both preoperative and postoperative chemotherapy was 75.8%, while it was 40.3% in those who received only preoperative treatment [20]. In another study with 299 patients, completion of adjuvant chemotherapy did not show a survival benefit in all patients [21]. However, in our study, completion of adjuvant chemotherapy was shown to have a DFS benefit (p = 0.038). The ratio of these patients is 56.8% of the whole study group.

Median number of preoperative and adjuvant FLOT cycles are 4 (range: 1–12) and 4 (range: 0–8), respectively. Fifteen percent (66) of patients received more than 4 cycles of FLOT as neoadjuvant. The R0 resection rate in these patients was 69.7%, significantly lower than in other patients. Twenty-six patients received more than 4 cycles of FLOT as adjuvant therapy. Although there is insufficient data for the prolonged therapy, these patients were probably more advanced and had a higher tumor burden. In the data, it was observed that there were patients with suspected metastasis at baseline and also inoperable after 4 cycles of neoadjuvant therapy and continued treatment.

HER-2 status, tumor location, disease stage, albumin and hemoglobin values, and tumor marker levels did not have any effect on survival, consistent with the literature.

Radiotherapy is used preoperatively and postoperatively in gastric cancers, especially in GEJ tumors. Although there is no head-to-head study of perioperative chemotherapy and adjuvant chemoradiotherapy, radiotherapy is not recommended in patients treated with perioperative chemotherapy unless R1–2 resection is performed. In this study, no difference in survival was found in patients who received adjuvant radiotherapy compared to those who did not.

The tolerability and safety profile of the FLOT were favorable than the clinical trial. The most common AEs were hematological (neutropenia and anemia) in both our study and the FLOT4-AIO trial. The most common grade 3–4 side effect was neutropenia with 11.8% in our study and 51% in the landmark study [3].

This study has several limitations. First, the follow-up period of the patients included in the study is short because FLOT regimen commonly used only in last few years. Therefore, some of the survival data are still immature but early results of this regimen in real-life is so important to accept as a standard regimen. Second, some data may have been missed because the clinical data were obtained from hospital records. Although it was understood from the patient files that there was no active infection, mild infection may not have been noted. Therefore, this may affect the ratio of NLR and PLR. Third, there is no antiplatelet agent treatment information that may affect platelet count and activity (It can affect the PLR). Fourth, there is no information about steroid therapy (may affect the blood count); however, this may cause minimal error because pretreatment steroid use is not common in Turkey. Fifth, since it is a multicenter study, surgery was performed by different clinics. This may also have affected the results. Finally, there is no information about microsatellite instability status of patients.

In conclusion, real-life perioperative FLOT regimen in operable gastric and GEJ tumors showed similar oncologic outcomes compared to clinical trials. Better performance status, receiving adjuvant chemotherapy as same regimen, lower grade and lower NLR and PLR improved outcomes in real-life.

## Figures and Tables

**Table 1:** Patients, disease and treatment characteristics.

	No.	% (n = 441)
Median age of diagnosis, years (range)	60 (18–85)	
Sex		
Female	145	32.9
Male	296	67.1
Location of primary tumor		
Gastroesophageal junction	204	46.3
Corpus	116	26.3
Antrum	106	24
Fundus	15	3.4
ECOG Performance Score		
0–1	398	90.2
2	11	2.5
Unknown	32	7.3
cT-N stage	338	
T1/T2 N (−)	3	0.9
T1/T2 N (+)	16	4.7
T3/T4 N (−)	15	4.4
T3/T4 N (+)	304	89.9
Signet cells		
Yes	124	28.1
No	218	40.4
Missing	99	22.5
Grade		
Grade 1	32	7.2
Grade 2	137	31.1
Grade 3	169	38.3
Undifferentiated	18	4.1
Missing	85	19.3
HER2 status	262	
Positive	15	5.7
Negative	247	94.3
The median cycle of neoadjuvant FLOT, range	4 (1–12)	
The median cycle of adjuvant chemotherapy, range	4 (0–8)	
FLOT	298	89
5-FU and -platin	16	4.8
5-FU	6	1.8
Others	15	4.4
Adjuvant radiotherapy		
Yes	75	17
No	366	83
Surgery		
Yes	413	93.7
No	28	6.3
Pretreatment median hemoglobine, gr/dL, IQR	12 (10.6–13.7)	
Pretreatment median NLR, IQR	2.8 (1.96–3.90)	
≥2.8	116	50
Pretreatment median PLR, IQR	167.7 (119.2–236.3)	
≥167.7	117	50.4
Pretreatment median CEA, ng/mL, IQR	2.6 (1.18–8.43)	
Pretreatment median CA19-9, U/mL, IQR	15.5 (6.4–54)	
Pretreatment median albumin, mg/dL, IQR	3.9 (3.6–4.2)	
<3	8	3.5

**Table 2:** Effect of variables on OS and DFS.

	Median DFS (95% CI), months	p value	Median OS (95% CI), months	p value
Median NLR				
Low	22.9 (19.9 to 25.9)	**0.039**	NR (NR to NR)	**0.007**
High	16.2 (12.4 to 20)		23.5 (17.1 to 29.9)	
Median PLR				
Low	23 (13.6 to 32.4)	0.062	NR (NR to NR)	**0.033**
Hihg	17.7 (16 to 19.5)		24.2 (21 to 27.4)	
HER2 status				
Positive	22.9 (7.2 to 38.6)	0.440	25.9 (9.9 to 41.9)	0.317
Negative	20.7 (16.2 to 25.3)		NR (NR to NR)	
ECOG performance score				
0–1	23.5 (18.8 to 28.3)	**0.019**	NR (NR to NR)	**0.001**
2	5.8 (0 to 13)		10 (5.8 to 14.2)	
Location of primary tumor				
Gastroesophageal junction	25.2 (18.8 to 31.6)	0.565	NR (NR to NR)	0.445
Corpus	22.9 (NR to NR)		NR (NR to NR)	
Antrum	21.7 (13.2 to 30.1)		29.2 (20.8 to 37.5)	
Fundus	20.7 (14.1 to 27.4)		NR (NR to NR)	
cT-N stage				
T3/T4 (N+)	20.7 (16.8 to 24.7)	0.184	29.2 (NR to NR)	0.192
Others	NR (NR to NR)		NR (NR to NR)	
Grade				
Grade 1	NR (NR to NR)	**0.001**	NR (NR to NR)	**0.018**
Grade 2	NR (NR to NR)		NR (NR to NR)	
Grade 3	18.1 (11.7 to 24.5)		28.7 (NR to NR)	
Undifferentiated	13.8 (3.5 to 24.1)		22.7 (12.8 to 32.5)	
Adjuvant chemotherapy				
FLOT	27.3 (NR to NR)	**0.012**	NR (NR to NR)	**0.004** 2
Others	17.4 (7.7 to 27)		NR (NR to NR)	
Adjuvant radiotherapy				
Yes	20.7 (19 to 22.5)	0.934	28.7 (19.7 to 37.6)	0.319
No	25.1 (19.5 to 30.8)		NR (NR to NR)	
Albumin, mg/dL				
<3	NR (NR to NR)	0.184	NR (NR to NR)	0.530
≥3	17.9 (14.5 to 21.4)		29.2 (21.6 to 36.7)	
CEA, >ULN^1^ (0–5), ng/mL				
Yes	27.2 (6 to 48.3)	0.946	NR (NR to NR)	0.163
No	19.1 (15.6 to22.5)		24.2 (19.9 to 28.4)	
CA19-9, >ULN (0–37), U/mL				
Yes	13.7 (8 to 19.4)	0.287	29.2 (7.8 to 50.5)	0.159
No	20.2 (17.2 to 23.2)		NR (NR to NR)	
Hemoglobine, gr/dL				
<10	NR (NR to NR)	0.337	NR (NR to NR)	0.608
≥10	18.1 (14.3 to 21.8)		29.2 (21.5 to 36.9)	

1 ULN: Upper limit normal,

2 In favor of FLOT.

**Table 3:** Response rates.

	No.	%
ORR (complete and partial response)	78	58.7
Complete response	13	9.8
Partial response	65	48.9
Stable disease	40	30.1
Progressive disease	15	11.3
pCR	52	12.9*

* Among patients who underwent surgery.

**Table 4:** The multivariate analysis of variables.

	Univariate analysis		Multivariate analysis	
	OR* (95% CI)	p value	OR (95% CI)	p value
NLR	2.21 (1.22–4.01)	**0.009**	**2.60 (1.07–6.36)**	**0.036**
PLR	1.8 (1.00–3.24)	**0.050**	1.21 (0.51–2.88)	0.667
Grade	2.04 (1.21–3.44)	**0.007**	1.08 (0.50–2.35)	0.849
CEA	0.54 (0.26–1.10)	0.091	0.48 (0.19–1.17)	0.107
CA19-9	1.26 (0.66–2.42)	0.485	1.54 (0.67–3.54)	0.311

* Odds ratio.

**Table 5:** The most common AEs during neoadjuvant and adjuvant treatment.

Adverse event	All grades, n (%)	Grade 3–4, n (%)
Any	57.6	23.6
Neutropenia	26.1	11.8
Anemia	19	0.9
Thrombocytopenia	10.2	1.1
Fatigue	9.5	1.6
Diarrhea	8.4	1.6
Neuropathy	8.2	1.4
Stomatitis	4.8	0.2
